# Microcystins in Aquatic Food Products: Linking Environmental Transfer, Consumed Matrices, and Dietary Risk

**DOI:** 10.3390/toxins18100439

**Published:** 2026-10-12

**Authors:** Xiaoyu Xiao, Junxuan Zeng, Songheng Zheng, Shifeng Liu, Qi Deng, Mei Qiu, Zhijia Fang, Gaoshuang Hu, Yaling Wang, Youxin Wang

**Affiliations:** 1 Guangdong Provincial Key Laboratory of Aquatic Product Processing and Safety, Guangdong Provincial Engineering Technology Research Center of Seafood, Guangdong Province Engineering Laboratory for Marine Biological Products, Guangdong Provincial Engineering Technology Research Center of Prefabricated Seafood Processing and Quality Control, College of Food Science and Technology, Guangdong Ocean University, Zhanjiang 524088, Chinaliusf927@stu.gdou.edu.cn (S.L.);; 2 Collaborative Innovation Center of Seafood Deep Processing, Dalian Polytechnic University, Dalian 116034, China; 3 College of Food Science and Biology, Hebei University of Science and Technology, Shijiazhuang 050018, China; 4 School of Public Health, North China University of Science and Technology, Tangshan 063210, China

**Keywords:** microcystins, aquatic food products, cyanobacterial blooms, bioaccumulation, dietary exposure, risk assessment, environmental governance

## Abstract

Microcystins (MCs) produced by cyanobacteria can contaminate aquatic foods, but drinking water surveillance does not directly characterize dietary exposure. This critical narrative and framework review evaluates environmental transfer, tissue residues, analytical coverage, toxicity, exposure assessment, and management for fish, bivalves, crustaceans, and related products, with a separate assessment of algal dietary supplements. Evidence is organized by species, consumed tissue, concentration basis, analytical fraction, congener coverage, and consumption scenario. Reported concentrations are not directly comparable when studies analyze different organs or use different extraction procedures and mass bases. Paired analyses of tilapia muscle illustrate how measurements limited to free congeners can miss a substantial additional burden, while shellfish studies show why clearance requires verification in the edible tissue. Structural and toxicological evidence explains why MC-LR provides a reference for assessment but does not fully characterize every mixture. We propose a framework linking environmental warnings to product sampling, validated quantification, explicit exposure scenarios, and proportionate management. Priorities are comparable occurrence reporting, interlaboratory validation, characterization of the oral relevance of bound fractions, and congener-specific toxicity data. Neither a positive screening result nor an exceedance of a chronic intake benchmark alone establishes the probability of illness from an individual meal.

## 1. Introduction

Microcystins (MCs) become food hazards when cyanobacterial material or dissolved toxins enter harvested organisms. Global risk management reviews distinguish environmental surveillance from assessment of human exposure [1,2]. For food, the relevant exposure depends on what is eaten, how much is consumed, and which toxin forms are measured, as emphasized in the EFSA evidence review [3] and the recent synthesis by Drobac Backović and Tokodi [4]. Aquatic foods must therefore be assessed as consumed products rather than as proxies for water quality.

The evidence base spans aquatic ecology, analytical chemistry, toxicology, exposure assessment, and regulation. Schreidah et al. reviewed MC biogenesis, toxicity, analysis, and control [5], whereas Abdallah et al. considered cyanotoxin contamination across food categories [6]. These complementary perspectives establish the hazard but also reveal a practical difficulty: environmental occurrence, tissue concentration, and a toxicological reference value answer different questions. Combining them without a defined matrix and exposure scenario can overstate or understate consumer risk.

This review centers dietary interpretation on the consumed matrix and separates environmental occurrence, biological uptake, tissue distribution, analytical validity, exposure, and management. It covers finfish, bivalves, crustaceans, small whole fish, products containing viscera, and related aquatic foods in which MC exposure may be relevant. Environmental signals are treated as sampling triggers rather than product residues, and hazard evidence is treated as context for risk characterization rather than a substitute for exposure data.

### 1.1. Review Scope and Literature Selection

This critical narrative and framework review evaluated the extent to which published evidence linked environmental microcystin occurrence to a defined aquatic product, an analytically characterized residue, an explicit dietary exposure scenario, and a management decision. Evidence was classified as occurrence and transfer, toxicology and exposure, analytical performance, processing and control, or regulatory guidance because each category supports a different inference.

The literature search covered database inception through 18 June 2026 in Web of Science Core Collection, Scopus, PubMed, Crossref, and OpenAlex, with official agency portals and citation tracking used for verification. A further update was conducted on 8 August 2026 without renewed exports from Web of Science or Scopus. The original exports and database attribution of selected records were not retained; historical selection counts by database and publication year cannot therefore be reconstructed reliably. Supplementary Table S1 distinguishes the documented search history from reproducible strategies for future updates. A documented PubMed search conducted on 2 October 2026 used separate searches for recent reviews, algal supplements, and recent tissue studies. These searches retrieved 68, 198, and 21 records, respectively, comprising 282 unique PubMed records after deduplication by PMID. These are retrieval counts for the PubMed searches, not counts of studies selected for the review. Supplementary Table S2 provides the publication year distribution and identifies records from these searches that are cited in this article.

Records were deduplicated by DOI matching followed by normalized title, first author, and publication year. The documented PubMed searches were used to identify directly relevant reviews and primary studies, followed by verification of the claims retained in this article. The search strategy was not designed as a systematic review, and no PRISMA flow diagram or claim of complete coverage is made.

Evidence was included when it linked an aquatic organism, food, or algal dietary supplement to an interpretable tissue or product definition, analytical fraction, concentration basis, method performance, exposure estimate, or management decision. Algal supplements were considered separately because biomass powders, capsules, and tablets differ from animal tissues in composition and consumption. Studies limited to water were used only to clarify environmental transfer, sampling triggers, or treatment mechanisms. Terrestrial foods, duplicate datasets, abstracts without sufficient primary detail, and outcomes unrelated to MCs were excluded from conclusions about aquatic products. Evidence was appraised qualitatively for directness to the consumed product, analytical suitability, reporting completeness, and decision relevance. No pooled prevalence or concentration estimate was calculated.

### 1.2. Contribution Relative to Recent Reviews

Recent reviews address several parts of this problem. Drobac Backović and Tokodi evaluated exposure and health effects across foods [4], while Drobac Backović and Tokodi examined cyanobacteria and cyanotoxins in freshwater aquaculture [7]. Fraga et al. systematically assessed transfer from freshwater into transitional and marine environments [8]; Villalobos et al. discussed health and environmental effects across these settings [9]. Shao et al. considered food chain transfer, detection, and detoxification [10]. These reviews provide context for interpreting contamination, but their distinct scopes do not supply an interchangeable set of residue and consumption data.

The update also identified reviews that strengthen the chemical and toxicological context. Melaram et al. considered the human health implications of microcystins and nodularin [11], and Rajpoot et al. reviewed MC-LR chemistry, exposure routes, mechanisms, and target organs [12]. Withana et al. examined microalgal toxins in food products, including products marketed for nutritional use [13]. A recent systematic review by Moon et al. assessed chronic human health outcomes but found that the quantitative evidence remained sparse or heterogeneous across endpoints [14]. Table 1 places these contributions alongside the present focus on the sequence from an identified food residue to a defensible consumer risk assessment.

The contribution of this review is to examine the compatibility of evidence used at each step in a product decision. It does not claim that transfer, toxicity, or analysis has been overlooked previously. Instead, it evaluates whether tissue identity, analytical coverage, concentration basis, oral hazard evidence, and consumption assumptions support the inference being made. This approach builds on earlier reviews of accumulation in freshwater foods [15,16].

Evidence is interpreted according to the inference supported by its design. Environmental studies inform transfer and sampling triggers; tissue studies establish residue occurrence; method studies establish analytical fitness; toxicology informs reference values; and regulatory documents define the decision context. This allocation prevents conclusions from being transferred across incompatible evidence types.

Section 2, Section 3, Section 4, Section 5 and Section 6 follow this evidential sequence from occurrence and analytical validity to exposure, control, and governance.

### 1.3. Chemical Structure and Congener Diversity

MCs are cyclic heptapeptides with the general sequence cyclo-(D-Ala^1^–X^2^–D-MeAsp^3^–Z^4^–Adda^5^–D-Glu^6^–Mdha^7^). X and Z are variable L-amino acids; D-MeAsp is D-erythro-β-methylaspartic acid, and Mdha is N-methyldehydroalanine. Adda is the characteristic hydrophobic β-amino acid, (2S,3S,8S,9S)-3-amino-9-methoxy-2,6,8-trimethyl-10-phenyldeca-4,6-dienoic acid. Variations include substitutions at positions 2 and 4 and modifications elsewhere in the ring, including demethylation [17].

The suffix identifies the residues at positions 2 and 4: MC-LR contains leucine and arginine; MC-RR contains two arginine residues; and MC-YR contains tyrosine and arginine. MC-LA, MC-LF, and MC-LW contain leucine paired with alanine, phenylalanine, and tryptophan, respectively. These names do not describe every structural modification; a demethylated congener requires additional positional specification where known [17]. Structural variation can affect phosphatase interaction and transporter affinity [18,19]. The chemical diversity therefore matters both to the calibration panel used for food analysis and to the interpretation of toxicity in Section 4.

## 2. Occurrence, Transfer, and Exposure in Specific Products

Occurrence evidence is organized by product and tissue rather than combined into a single concentration distribution. The quantitative examples in Table 2 retain the reported analyte and mass basis. This allows differences relevant to consumption to be examined without treating internal organ burdens, edible muscle, whole soft tissues, and algal powders as equivalent samples.

### 2.1. Evidence Characteristics and Reporting Limitations

The evidence is uneven in its direct relevance to food. Preece et al. documented MCs in a benthic food web [20], while their earlier study connected freshwater sources to residues in marine mussels [21]. Straquadine et al. demonstrated accumulation in oysters and clams after exposure to *Microcystis* [22]. More recent field studies describe seasonal or interannual variation in estuarine shellfish and cultured oysters [23,24]. These studies establish plausible pathways and variable occurrence; their sampling designs do not estimate the prevalence of contamination in the global seafood supply.

Analytical fraction is an additional source of heterogeneity. Greer et al. reported mean tilapia muscle concentrations of 15.45 µg/kg dry weight for free MC-LR and 110.1 µg/kg dry weight for total MCs measured after oxidation [25]. Pham et al. likewise distinguished free and covalently bound fractions in fish and bivalves [26]. The difference between these operational measurements is evidence of incomplete coverage by a restricted assay, not proof that every additional microgram has the oral potency of free MC-LR.

Tissue comparisons also change interpretation. Jia et al. reported 31.7 ± 12.1 µg/kg dry mass in common carp muscle, whereas the intestinal wall of crucian carp contained 2040 ± 4430 µg/kg dry mass [27]. These results concern different species and organs and cannot be treated as a partition coefficient within one animal. Wilson et al. compared yellow perch muscle and liver [28], and Peng et al. evaluated fish and shellfish from three Chinese lakes [29]. Together, these studies support recording tissue identity and local consumption practices rather than applying a universal concentration ratio.

For this review, a concentration is interpretable only when the source specifies the species or product, tissue, analyte or analytical fraction, unit, and mass basis. Sampling location, design, recovery, detection limits, and consumer relevance are retained where reported. Table 2 presents selected quantitative examples, not a ranked comparison of species or a pooled estimate of dietary risk.

**Table 2:** Selected measured microcystin concentrations with the original tissue and reporting basis retained.

Source and Design	Species or Product	Analyte and Method	Measured Concentration and Basis	Consumer Interpretation
Greer et al. [25] Aquaculture survey	Tilapia muscle	Free MC-LR by LC-MS/MS; broader MC burden after oxidation	Means: free MC-LR 15.45 µg/kg dw; total MCs 110.1 µg/kg dw	A restricted free assay can miss additional burden; fresh serving estimates need moisture data
Jia et al. [27] Lake Taihu field study	Common carp *Cyprinus carpio* Muscle	MC-LR, MC-RR, and MC-YR by LC-MS/MS	31.7 ± 12.1 ng/g dry mass (=µg/kg dw)	Directly relevant tissue for filets, but still requires a measured moisture conversion
Jia et al. [27] Lake Taihu field study	Crucian carp *Carassius auratus* Intestinal wall	MC-LR, MC-RR, and MC-YR by LC-MS/MS	2040 ± 4430 ng/g dry mass (=µg/kg dw)	An internal organ result; not a filet concentration or a serving estimate
Camacho-Muñoz et al. [30] Controlled uptake study	Marine mussel *Mytilus edulis* Whole soft flesh	Individual congeners by LC-MS/MS	At day 7: MC-LR 0.74; MC-LF 0.35; MC-LW 0.31 µg/g fresh flesh	Laboratory accumulation, not retail prevalence; reported combined MC/nodularin burden is a different quantity
Trung et al. [31] Vietnam field samples	Whiteleg shrimp *Litopenaeus vannamei* Whole head	Extractable MC congeners by LC-MS/MS	15.21 µg/g dw in the positive head sample	Relevant where heads are consumed; cannot be applied to peeled tail muscle
Trung et al. [31] Vietnam field samples	Apple snail *Pomacea canaliculata* Muscle	Extractable MC congeners by LC-MS/MS	0.15–0.30 µg/g dw in positive muscle samples	Concentration in positive samples is not a population mean or prevalence estimate
FSA [32] Lough Neagh assessment	Perch *Perca fluviatilis* Edible flesh composites	Total MCs and nodularins	21/23 composites <5 µg/kg ww; two at 6.6 and 61 µg/kg ww	Includes nodularins; assessment considered variability and concluded no health concern under its scenarios
Van Hassel et al. [33] Belgian market survey	Algal powders, capsules, and tablets	Targeted MC congeners by LC-MS/MS	238.45–5645.33 µg/kg marketed product in eight quantified samples	Use product mass taken per day; not a fresh tissue or generic dw comparison

Note. dw, dry weight or dry mass; ww, wet weight. The symbol ± denotes the variability reported by the source, not a common uncertainty model across rows. No generic moisture conversion is applied. For Camacho-Muñoz et al., the fresh basis follows the extraction of weighed homogenized fresh flesh described in the methods. For Trung et al., shrimp whole head is the designation in the summary table; the main summary table calls this visceral mass. The FSA measurement includes nodularins and is not a congener sum restricted to MCs. Values from laboratory exposure, field samples, and commercial products are kept distinct.

### 2.2. Environmental Transfer as a Product Safety Pathway

Nutrient supply, temperature, hydrology, and cyanobacterial composition influence bloom development and the timing of toxin exposure [34]. MCs can occur in intact cells, dissolved water, particles, and material entering food webs. Brown et al. describe how harmful blooms affect mariculture and fisheries [35]. The FAO, IOC, and IAEA technical guidance recommends integrating observations and laboratory information within early warning systems [36]; in this review, such warnings are used to prioritize product sampling.

A bloom can justify precautionary restrictions while product evidence is being collected, particularly where exposure is plausible and testing is delayed. Its presence does not, by itself, quantify the dose in an edible portion. Conversely, one nondetect does not establish that a harvest area or batch is free from contamination. Decisions require sampling appropriate to the affected area and a documented basis for restriction, reassessment, and release, consistent with the planning principles in harmful bloom monitoring guidance [36,37].

Transfer through cells, particles, and food webs connects freshwater sources with downstream harvest areas, as reviewed by Preece et al. [38]. Bukaveckas et al. documented MCs in Baltic and Chesapeake Bay food webs [39], and Zhang et al. examined transfer and distribution in Lake Taihu [40]. These observations support several exposure routes, but they do not establish a single transfer coefficient applicable to fish, filter feeders, and benthic organisms.

Flores et al. found variable relationships between MC concentrations in water and fish across studies [41]. Spatial and temporal variation in fish contamination was also documented in the Lake Chaohu basin [42]. A water sample therefore describes environmental conditions at a particular place and time, whereas a tissue sample integrates uptake, distribution, and elimination. We recommend coordinated environmental and tissue sampling when the purpose is to identify an exposure window or verify clearance.

### 2.3. Tissues, Edible Portions, and Reporting Basis

Fish organ burdens can differ markedly from residues in edible muscle, as shown in tilapia and several freshwater fish species [43,44]. In crayfish, Miles et al. found substantial variation in congener profiles and an important contribution from a metabolite not represented by a restricted target panel [45]. Bivalve studies add a different matrix because the whole soft body is often relevant to consumption [46]. The sampled organ and the edible portion must therefore be identified explicitly; a high liver or intestinal concentration cannot be substituted for a filet concentration.

Concentrations on a wet-weight basis refer to the mass of hydrated tissue, whereas dry-weight concentrations refer to the mass remaining after drying. Conversion requires the measured dry matter fraction: *C_ww_* = *C_dw_* × (dry mass/wet mass). The dry mass values reported for fish in Table 2 are therefore retained as such [25,27]. Without moisture data for the relevant tissue, converting them to concentrations in a fresh serving would introduce an unquantified assumption.

Preparation determines which anatomical compartments remain in the food. Fileted muscle, whole bivalve soft tissue, shrimp heads, and dishes retaining viscera are distinct samples. Heating can also redistribute MCs between tissue and cooking liquid, as examined in fish processing experiments [47]. Reports need to identify whether the sample represents the raw edible portion, the cooked food, or both food and retained cooking liquid. Quantities consumed and the frequency of consumption are considered separately in Section 4.3.

**Figure 1:**
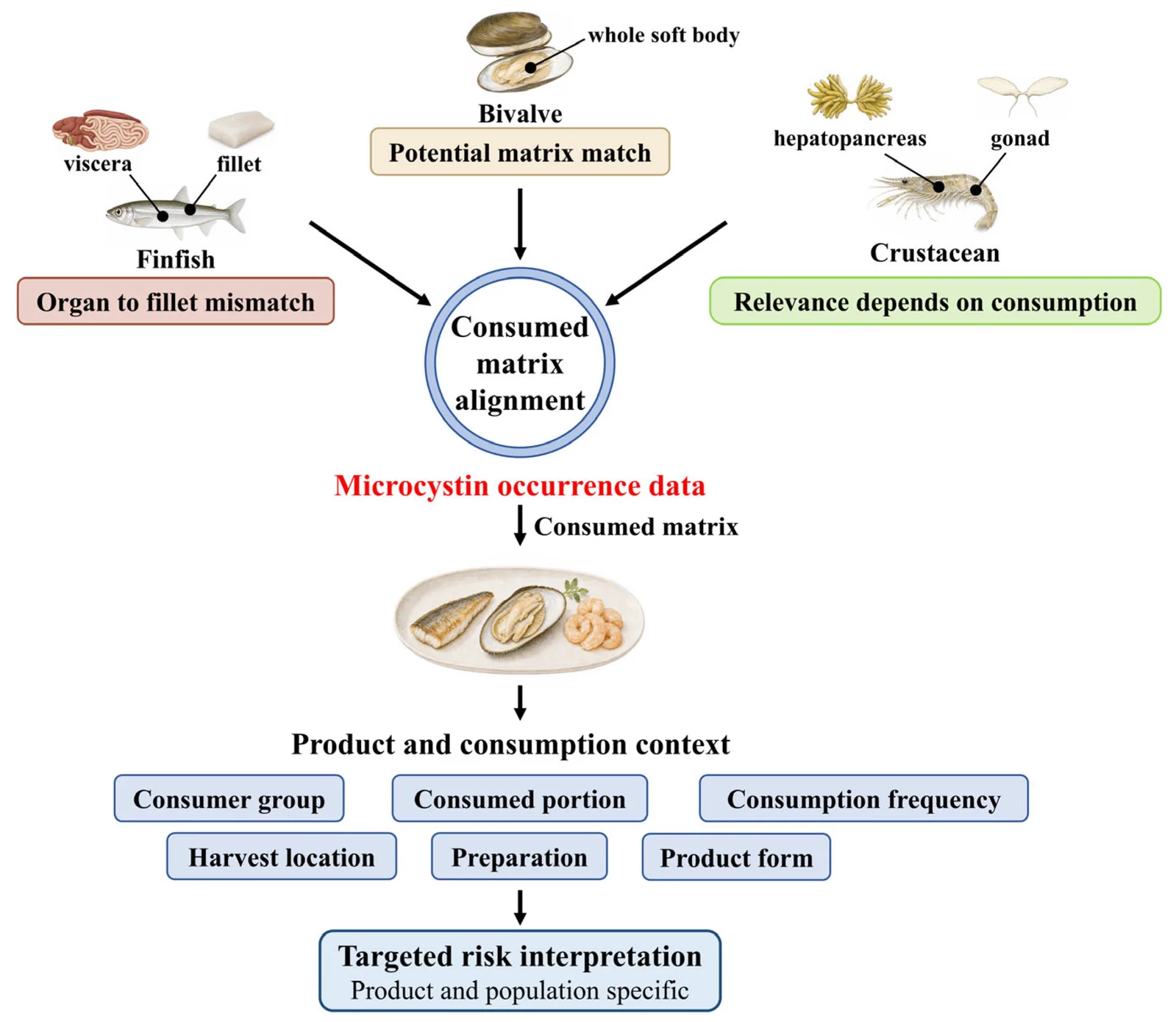
Alignment of measured and consumed matrices in microcystin assessment for aquatic products. Residue interpretation depends on whether the analyzed tissue matches the consumed product and on product-specific preparation and consumption assumptions.

The Food Standards Agency Lough Neagh assessment [32] separated edible flesh from liver and gastrointestinal tissue and evaluated incomplete evisceration and preparation of whole fish. Its transferable value for this section is the explicit alignment of analyzed tissue, product form, and reporting basis, not the concentration or advisory outcome at that site (Figure 1).

### 2.4. Free, Bound, Masked, and Total MC Fractions

Analytical fractions require operational definitions. Free MCs are parent congeners recovered by the stated extraction procedure. Bound fractions can include covalent associations with tissue components, whereas masked is a broader description of material not recovered by a particular assay. Oxidation of the Adda moiety to 2-methyl-3-methoxy-4-phenylbutyric acid (MMPB) estimates a broader burden but removes information on the original congener [48]. Consequently, total MC is a method definition, not a guarantee that every related compound has been measured.

Each analytical fraction serves a different purpose. Targeted LC-MS/MS confirms specific congeners; methods based on oxidation estimate a broader burden containing Adda; immunoassays and functional assays primarily support screening. A methodological review of total MC forms in biological matrices identifies the same distinction between broader burden estimates and measurements that distinguish individual congeners [48]. Reports intended to support decisions require the tissue, extraction procedure, analytical fraction, unit basis, and intended role as screening or confirmation.

Lance et al. demonstrated accumulation of free and covalently bound MCs in a freshwater snail model [49]. Kondo et al. characterized glutathione and cysteine conjugates [50], and Wu et al. measured these conjugates in common carp tissues [51]. Such findings support analytical expansion beyond parent congeners, but neither detection nor chemical mass alone establishes bioavailability after ingestion. Incorporation into a toxicity equivalent calculation requires evidence on digestion, absorption, and relative oral potency.

Paired free and total measurements can identify incomplete analytical coverage, as illustrated by the tilapia data [25]. Their difference is not a universal correction factor for another species or tissue. Oxidation recovery, the specificity of the measured Adda product, congener composition, and the biological relevance of the additional fraction remain separate uncertainties [48].

### 2.5. Populations Requiring Distinct Exposure Assessment

The occurrence literature does not establish a single risk estimate for all consumers. Fishers relying on local harvests may encounter a different sequence of products and exposure periods from occasional consumers, as discussed for communities in Winam Gulf, Lake Victoria [52]. Section 4.3 translates this distinction into explicit assumptions about portion size, consumption frequency, and body weight.

Susceptibility is also distinct from the amount consumed. Lin et al. reported an association between serum MC-LR and gestational diabetes in a nested case–control study [53]. This observation does not identify contaminated seafood as the exposure source or establish a causal effect in pregnant consumers. Evidence concerning repeated exposure and inflammation provides additional hazard context [54] but does not justify assigning a numerical susceptibility factor to a population.

We therefore recommend presenting scenarios for children and frequent local consumers separately, while describing pregnancy and existing disease as uncertainties requiring more direct evidence. Population labels alone cannot establish dose or the probability of harm.

### 2.6. Potential Combined Exposure Modifiers

Microplastics are a potential modifier rather than an established quantitative component of dietary MC risk [55]. Moura et al. demonstrated adsorption of eight MC analogs to plastic polymers [56]. Controlled zebrafish studies reported altered hepatic effects during combined exposure to MC-LR and plastic particles [57,58]. These findings support mechanistic plausibility, but differences in particle condition, dose, species, and tissue distribution prevent direct application of a multiplier to harvested food. Combined exposures can be recorded in the uncertainty assessment without assuming a known effect on consumer dose.

### 2.7. Algal Dietary Supplements as a Distinct Exposure Source

Algal powders, tablets, and capsules marketed as natural products are relevant to MC exposure but cannot be treated as fish or shellfish tissues. Withana et al. discuss microalgal toxins in nutritional products [13]. Contamination also needs to be distinguished from toxin production by the intended ingredient: genomic analysis of *Limnospira* used for spirulina found no genes for MC synthesis in the examined genomes [59]. A contaminated commercial product is therefore not evidence that the marketed organism itself produced the toxin.

Van Hassel et al. detected MCs in 9 of 35 algal products from the Belgian market; eight products had quantified concentrations ranging from 238.45 to 5645.33 µg/kg of marketed product [33]. Miller et al. found some algal supplements containing more than 40 µg/g by mass spectrometry, with different numerical responses from immunochemical and inhibition assays [60]. In a subsequent Chinese survey, Liu et al. detected one or more of four MC congeners in 7 of 19 products using a method validated for capsules, liquids, powders, and tablets [61]. These are findings in the sampled products, not estimates of contamination in every brand or market.

Carbonell-Rozas et al. combined targeted analysis with suspect screening to broaden congener identification in spirulina and klamath supplements [62]. We recommend recording ingredient identity, formulation, batch, product concentration, target panel, and the daily mass consumed. The 1 µg/g value used for comparison in some supplement studies is not a universal statutory limit for all foods [60,63]. Risk interpretation needs the applicable jurisdiction and an explicit intake calculation, as shown in Section 4.3.

## 3. Analytical Methods and Decision Roles for Aquatic Products

These occurrence data are comparable only when analytical coverage and reporting basis are aligned. The following section therefore examines uncertainty arising from analytical methods. For aquatic products, decision suitability depends on sampling, extraction, analyte definition, matrix validation, quality control, and a predefined confirmation pathway; analytical sensitivity alone is insufficient [64,65]. Table 3 summarizes the roles and validation conditions of the principal method classes.

### 3.1. Sampling and Extraction as the First Analytical Decision

Sampling determines whether a result represents an individual, a composite, a harvest area, or a market batch. The FSA assessment illustrates the importance of this distinction by analyzing composite edible flesh samples separately from internal organs [32]. Extraction then defines the measured fraction. We recommend documenting dissection, homogenization, storage, and subsampling before comparing concentrations, because otherwise sampling variation can be mistaken for analytical or biological variation.

Nondetects require an explicit limit and cannot be treated as proof of absence. In an exposure calculation, lower and upper estimates can be explored by substituting zero and the relevant reporting limit, provided the rule is stated consistently. A detected value below the LOQ is also distinct from a value below the detection limit. The FSA assessment demonstrates how reporting limits and sample variability affect interpretation of edible tissue results [32].

Fish and shellfish methods provide direct evidence of matrix dependence. Mekebri et al. validated LC-MS/MS for water and tissue [80], while Karlsson et al. examined matrix effects in quantitative tissue analysis [81]. Van Camp et al. developed and validated a fish method for environmental and food monitoring [66]. These studies support calibration and recovery checks in the actual tissue, rather than transferring performance from an aqueous standard.

Geis-Asteggiante et al. compared LC-MS/MS and ELISA results in fish [82]. Haddad et al. applied isotope dilution to simultaneous cyanotoxin analysis [83], whereas Kohoutek et al. showed why chromatographic and mass spectrometric selectivity matter when estimating tissue concentrations [84]. Extraction studies using fish tissue and MC-LR conjugates further demonstrate that the recovered analyte set depends on the procedure [85,86]. A recent method covering several food matrices reported validation in aquatic products and algal supplements, reinforcing the need to retain matrix-specific recoveries and reporting limits [87].

For quantitative use, we recommend procedural blanks, fortified matrix samples, replicate extraction, appropriate calibration, and internal standards where available. The validation studies in fish [66] and shellfish or algal powders [67] provide practical examples. Batch acceptance criteria must define how contamination, inadequate recovery, or signal drift is handled. The development of a mussel matrix reference material also provides a basis for comparing laboratories with material that more closely represents the sample than a solvent standard [88].

### 3.2. Staged Workflow: Screening, Confirmation, and Total Burden

Targeted LC-MS/MS is suitable for congener quantification when identity criteria, calibration, extraction recovery, and batch quality control are established for the matrix. Turner et al. validated a method for shellfish and algal supplement powders [67]; Huang et al. subsequently reported validation for marine and estuarine shellfish [68]. Coverage remains limited by available standards. LC-HRMS can extend identification, as demonstrated for novel MCs in extracts [69] and for targeted and untargeted profiling in catfish [70], but tentative assignments must remain distinct from calibrated concentrations.

ELISA measures antibody recognition rather than a chemically resolved congener sum. Preece et al. assessed its use in seafood [71], whereas EPA Method 546 was developed for water and cannot by itself establish performance in edible tissue [72]. Recent evaluation against 19 reference materials improved understanding of congener cross-reactivity and included crayfish stomach samples [89]. Nevertheless, a screening value remains dependent on the antibody, extraction, and calibration used. Protein phosphatase inhibition assays measure a functional response; Garibo et al. compared inhibition equivalency among variants [73], and Alba Posse et al. validated an optimized assay [90]. Neither format independently identifies every compound contributing to the signal.

Oxidation followed by MMPB measurement addresses a different question: whether a broader burden containing Adda is present. Anaraki et al. developed an approach for fish tissue [75], and Bouteiller et al. examined total MC measurement in fish and mussels [76]. Earlier tissue studies also distinguished total and covalently bound forms [77]. The result complements congener analysis but does not preserve the original mixture profile. Discordant results therefore require investigation of recovery, cross-reactivity, unmeasured congeners, and bound fractions rather than automatic substitution of one assay result for another.

A staged workflow can direct analytical effort to the consequence of the decision. Lateral flow assays evaluated for fish [78] and immunochromatographic analysis of water and fish [79] support rapid prioritization. In contrast, optical sensors developed for water [91] and devices tested in water or simulated body fluids [92] do not establish fitness for seafood without additional validation. We propose using a matrix-validated screen or targeted analysis first, followed by quantitative confirmation where the result may support an advisory or market action. Additional total burden analysis is a separate decision, informed by the suspected analytical gap.

## 4. Toxicity and Dietary Risk Interpretation

**Figure 2:**
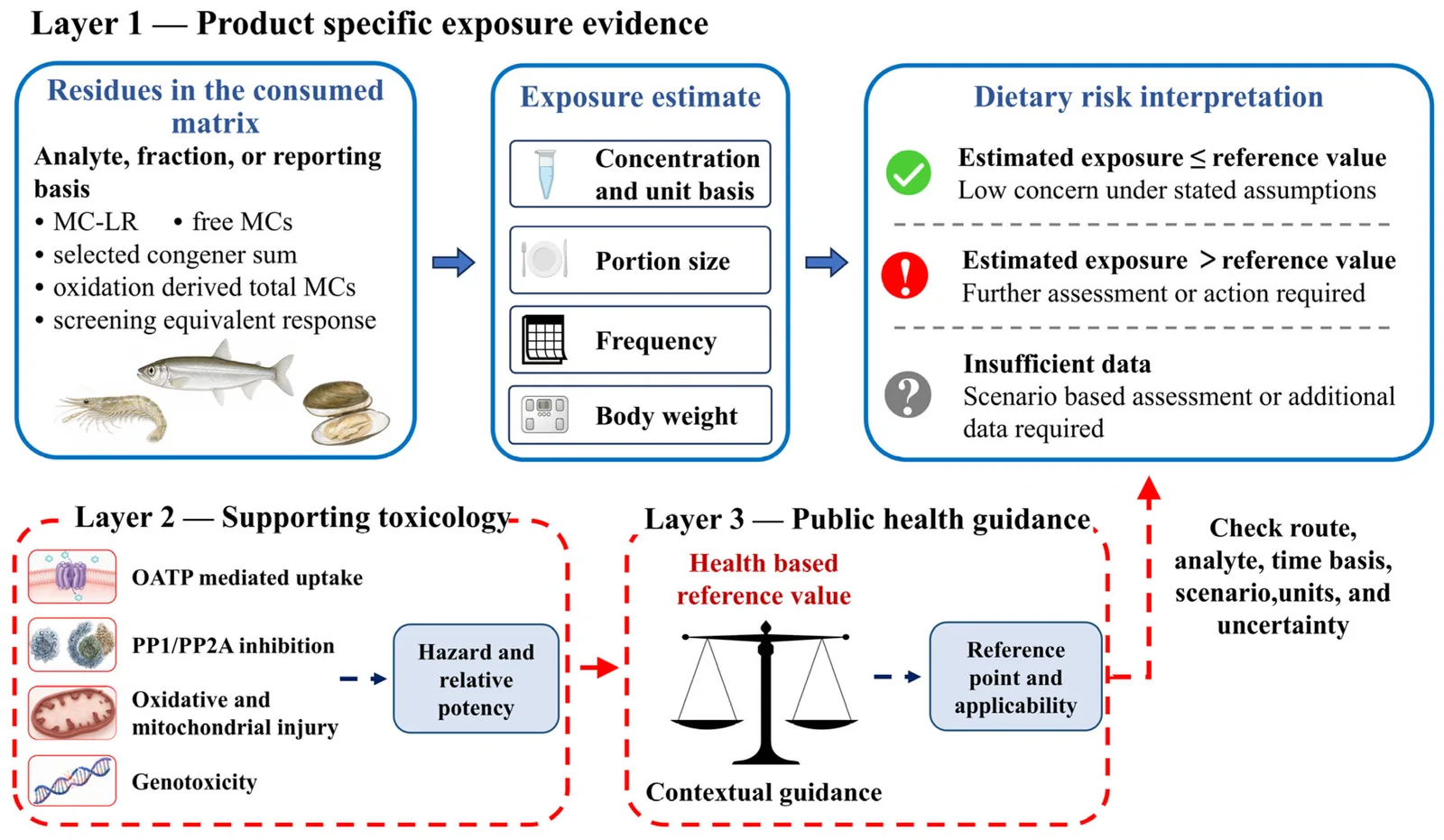
Evidence hierarchy for dietary risk characterization of microcystins in aquatic products. Residues measured in the consumed matrix provide the exposure input; toxicological evidence and toxicological reference values provide supporting context. MC-LR, microcystin-LR; MCs, microcystins; OATP, organic anion transporting polypeptide; PP1/PP2A, protein phosphatases 1 and 2A. The PP1–MC-LR topology was informed by RCSB PDB entries 1FJM and 6OBQ; 3FGA was used only as a PP2A scaffold, not as a microcystin-bound PP2A structure. The OATP pathway was informed by Fischer et al. [94].

Hazard and exposure are complementary inputs to risk characterization. Phosphatase inhibition establishes a molecular mechanism [93], while transporter studies explain part of the variation in cellular uptake [94]. Residue measurements and consumption assumptions determine the external dietary dose. Figure 2 summarizes how these distinct inputs contribute to an assessment; their combination must retain uncertainty about oral absorption and congener composition.

### 4.1. Toxicity Mechanisms and Limits of Extrapolation

MCs inhibit serine/threonine protein phosphatases, particularly PP1 and PP2A, as established by MacKintosh et al. [93]. Noncovalent interactions position the toxin in the catalytic region, and the Mdha residue can subsequently form a covalent linkage with a cysteine residue in the enzyme [18]. Inhibition disturbs protein phosphorylation and can disrupt cytoskeletal organization. Hooser et al. documented actin changes in hepatocytes [95], and Zeng et al. linked microfilament disruption to altered phosphorylation of associated proteins [96].

Oxidative stress and mitochondrial dysfunction contribute to downstream injury but are not interchangeable with phosphatase inhibition. Ding et al. reviewed oxidative mechanisms [97], and Weng et al. studied reactive oxygen species and apoptosis in hepatocytes [98]. Other experimental systems showed cytoskeletal disruption [99] and signaling changes in cells expressing OATP1B1 [100]. Woolbright et al. demonstrated predominantly necrotic injury in primary human hepatocytes [101]. Thus, the mode of cell death depends on the model and exposure conditions rather than constituting a single obligatory pathway.

Dietary extrapolation requires the route of exposure to be retained. The antioxidant response study by Moreno et al., for example, used intraperitoneal MC-LR administration [102], which bypasses the intestinal absorption barrier. Reviews of MC-LR toxicology also combine cellular experiments and animal studies with different routes and doses [12]. Such evidence identifies mechanisms and possible target organs but cannot directly predict the risk from cooked tissue containing a mixture of parent toxins and conjugates. Absorption, bioaccessibility, and repeated oral dose–response data remain necessary for that step.

OATPs influence intracellular exposure. Fischer et al. demonstrated transport by hepatic and brain OATPs [103], and subsequent work showed uptake that differs among congeners and toxicity in hepatocyte models [94]. Equal extracellular concentrations can therefore produce different intracellular doses. This helps explain why activity in a phosphatase assay without intact cells, cultured cells, and an intact organism need not yield the same potency ranking.

Genotoxicity is a further hazard consideration, but results from cyanobacterial extracts cannot automatically be assigned to MC-LR alone [104,105]. Human evidence also requires restraint. Moon et al. found that quantitative synthesis of chronic hepatic outcomes rested on two independent estimates, while renal and metabolic evidence was sparse or heterogeneous [14]. That review supports concern about repeated exposure but does not attribute a quantified disease burden specifically to aquatic foods. Water, supplements, and other exposure sources can confound an association based on a circulating biomarker.

### 4.2. Congener Mixtures and MC-LR Reference Assumptions

MC-LR is the main reference congener because its database supports widely used guidance, not because all other congeners have the same potency [106]. Ikehara et al. compared PP2A inhibition by 21 congeners [107], whereas Niedermeyer et al. demonstrated differences in OATP selectivity [19]. These are distinct determinants of toxicity. Neuronal studies [108,109] and intestinal uptake experiments comparing MC-LR with MC-RR [110] further show that biological context matters. Turco et al. used a human intestinal model to examine effects of different congeners [111]. A potency ratio from one endpoint cannot therefore be assumed to describe relative oral toxicity.

For a measured mixture, the assessment needs to identify the quantified congeners, any components estimated against a surrogate standard, and whether MC-LR potency is being assumed for the sum. A broader mass measurement is useful for detecting an analytical gap, but an assumption of equal potency is a screening convention rather than a validated conversion of every MC form to an equivalent oral dose [48,106].

**Figure 3:**
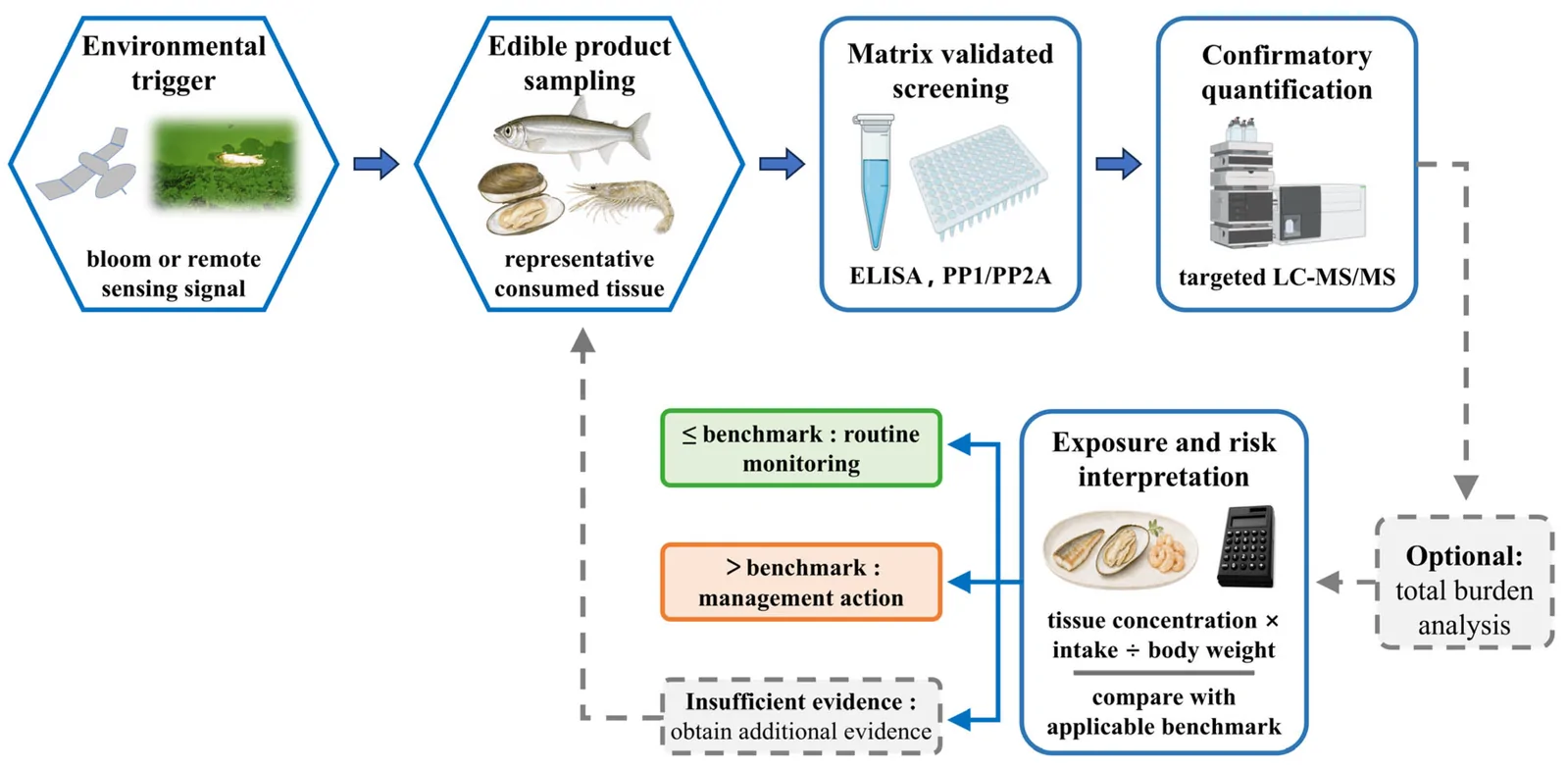
Framework linking evidence to governance for microcystins in aquatic food products. Environmental warnings lead to sampling, analysis, and interpretation of exposure. Total burden analysis is an optional, separately interpreted measurement. Green and orange boxes distinguish routine monitoring from management action; gray dashed boxes identify insufficient evidence and optional analysis. The written labels preserve these distinctions in grayscale. Dashed arrows indicate additional analysis or return for further evidence. MC, microcystin; LC-MS/MS, liquid chromatography coupled with tandem mass spectrometry. The framework is a proposal to support decisions, not a standardized regulatory protocol.

Toxic equivalency factors (TEFs) could eventually support calculation of toxic equivalents (TEQs), but their use requires a common endpoint, comparative oral dose–response data, and adequate analytical coverage. Structure–activity evidence does not yet resolve these requirements for all congeners and bound fractions in food [18]. We therefore treat TEF development as a research priority, not an established compliance procedure. The framework in Figure 3 can be implemented without assuming that such factors are already available.

### 4.3. Dietary Risk Characterization

Once analytical uncertainty has been characterized, tissue concentrations can be translated into exposure estimates under explicitly defined consumption scenarios.

$$\mathrm{EDI}\;\left(\mu g\;kg^{-1}bw\;day^{-1}\right)=\frac{C_{\mathrm{tissue}}\left(\mu g\;kg^{-1}ww\right)\times \mathrm{IR}\left(\mathrm{kg}\;day^{-1}\right)}{\mathrm{BW}\left(\mathrm{kg}\right)}$$

*C_tissue_* is the concentration in the consumed tissue (µg/kg ww); IR is the ingestion rate averaged over the stated exposure period (kg/day); and BW is body weight (kg). The abbreviations ww and bw denote wet weight and body weight. A long term EDI requires a concentration and consumption rate representative of repeated intake, including the frequency of consumption; a seasonal or maximum concentration is not automatically a lifetime average.

For a single meal, the acute dose is calculated as

$$D_{\mathrm{meal}}\left(\mu g\;kg^{-1}bw\right)=\frac{C_{\mathrm{tissue}}\left(\mu g\;kg^{-1}ww\right)\times IR_{\mathrm{meal}}\left(kg\;meal^{-1}\right)}{\mathrm{BW}\left(\mathrm{kg}\right)}$$

*D_meal_* is the dose from one meal (µg/kg bw), and *IR_meal_* is the mass consumed in that meal (kg). EDI and *D_meal_* answer different questions. Studies of fish consumption illustrate the need to retain local intake assumptions [112,113]. The chronic tolerable daily intake (TDI) used by WHO is 0.04 µg/kg bw/day, derived from MC-LR toxicity data with uncertainty factors [114]. It is not an acute toxicity threshold, and a single meal dose cannot be interpreted as a chronic exceedance.

An illustrative calculation shows how the scenario changes interpretation. At an assumed tissue concentration of 10 µg/kg ww, repeated consumption averaging 0.10 kg/day gives an EDI of 0.0167 µg/kg bw/day for a 60 kg adult and 0.0667 µg/kg bw/day for a 15 kg child. Under the explicit assumption that the measured concentration can be compared with the MC-LR TDI, the corresponding quotients are 0.42 and 1.67. These are constructed scenarios, not observed intake distributions or probabilities of illness. A 0.30 kg meal at the same concentration gives an adult meal dose of 0.050 µg/kg bw; that value requires an assessment appropriate to acute exposure, not comparison with the chronic TDI as though it were a daily intake.

The Lough Neagh assessment illustrates why isolated concentrations need context. Of 23 perch flesh composites, 21 were below 5 µg/kg ww for total MCs and nodularins; the other two were 6.6 and 61 µg/kg ww [32]. The FSA considered the unusually high result, variability, consumption patterns, and short-term guidance, and concluded that the assessed exposure did not present a health concern. The high value alone therefore cannot be represented as a general finding of unsafe perch, nor can the overall conclusion be transferred to other fisheries without matching data.

Supplements require the marketed product concentration and the recommended or actual daily product mass, rather than a seafood portion assumption. For example, 5645.33 µg/kg in an algal powder consumed at 4 g/day gives an estimated intake of approximately 0.323 µg/kg bw/day for a 70 kg adult, using a measured product and label dose from Van Hassel et al. [33]. This calculation assumes the quantified MC sum is compared with the MC-LR TDI; it is not a validated congener TEQ. It shows how repeated use of a small mass of concentrated product can merit attention, without characterizing all algal supplements as unsafe.

The assessment should identify the principal uncertainty before selecting the next action. An incomplete target panel calls for additional analytical coverage; a dry mass result without moisture data cannot support a fresh portion estimate; and uncertain consumption calls for a range of explicit scenarios. Probabilistic analysis can describe variability only when suitable distributions are available [115]. We recommend reporting the exposure estimate, benchmark, comparison assumptions, and uncertainty together, followed by a proportionate action such as confirmatory analysis, additional sampling, or a targeted advisory.

## 5. Processing, Prevention, and Control Interventions

Control measures act at different points in the exposure pathway. Environmental prevention and surveillance address exposure before harvest [35], whereas food processing studies examine residues after uptake [47,116]. Evaluation must distinguish reduced toxin mass from altered concentration caused by moisture loss, transfer to cooking liquid, or changed extractability. Evidence from treatment of water is considered separately because it does not demonstrate decontamination of edible tissue.

### 5.1. Prevention and Monitoring Before Product Contamination

Nutrient management addresses conditions favoring cyanobacterial blooms [34]. Remote sensing can support surveillance over large areas [117], and early warning systems integrate observations with response planning [118]. Forecasting work at Lake Müggelsee illustrates the need to validate predictions in the intended environment [119]. Warming and hydrological change can alter bloom timing and persistence [120,121]. These approaches support prevention and sampling decisions, but their benefit for food safety still requires verification in harvested products.

Aquaculture provides potential control points not always available in open fisheries. Reviews of recirculating systems describe opportunities and practical constraints in water management [122,123]. Site selection also influences shellfish exposure and production conditions [124]. Nutrient discharge from shrimp ponds and fish cages can contribute to local pressure [125,126]. We recommend assessing intake water, recirculation, harvest timing, and nutrient management together, while testing whether an intervention actually reduces residues in the marketed product.

### 5.2. Depuration, Cooking, and Processing

Depuration is species-dependent and may leave a persistent residue. Zebra mussel studies examined uptake and elimination [127,128], while Camacho-Muñoz et al. demonstrated rapid accumulation and slow clearance in marine mussels [30]. Accumulation and elimination have also been examined in freshwater shrimp [129] and crayfish [130]. More recent fish experiments reported persistence after a depuration period [131]. Consequently, release after holding requires measurements in the relevant edible portion; an elapsed waiting period or disappearance of the bloom is not sufficient evidence of clearance.

Thermal experiments with fish muscle demonstrate that temperature and analytical extractability can alter the measured MC concentration [116]. Boiling and microwave studies in fish [47] and processing experiments with edible clams [132] extend this evidence to food preparation. Chemical stability studies under different pH and temperature conditions provide mechanistic context but are not demonstrations of detoxification in a meal [133]. We recommend measuring tissue, retained cooking liquid, moisture, and the toxin fraction of interest together. Available evidence does not justify recommending household cooking as a reliable remedy for a known contaminated product.

### 5.3. Water Treatment and Degradation Technologies as Control Nodes

Water treatment technologies are relevant to source, production, or processing water, not automatically to contaminated food. Shawwa and Smith studied MC-LR reaction kinetics during ozonation [134]; Acero et al. evaluated chlorination kinetics [135]. Kim and Lee considered the toxicity implications of ozonation products [136], while Merel et al. characterized products generated by chlorination [137]. These distinctions matter because disappearance of a parent peak does not establish the safety of the treated mixture.

Ultraviolet and advanced oxidation studies address reaction pathways and treatment performance [138,139]. Biological alternatives include microbial degradation [140], MlrA enzyme systems [141], and treatment through biologically active sand filtration [142]. Such evidence supports evaluation of upstream controls. It does not establish that toxin already retained in fish muscle or shellfish soft tissue has been removed.

Selection of a water treatment process requires evaluation of the actual water composition, treatment dose, contact time, and transformation products. Reviews of advanced oxidation [139] and enzymatic degradation [141] identify constraints on effectiveness and implementation. For aquaculture, we recommend additionally verifying compatibility with the production system and confirming any claimed reduction in edible tissue residues. Treatment performance in a laboratory solution remains insufficient for that conclusion.

## 6. Regulatory Frameworks and Governance

Regulatory interpretation must distinguish guidance for health protection, enforceable limits, analytical standards, and assessments of a particular site. Guidance for drinking water addresses a different consumption pathway from food [143]. Concerns about extrapolating water guidance to cyanobacterial supplement products have also been discussed explicitly [63]. Table 4 separates the legal and technical roles of these documents.

Where no harmonized maximum level exists for an aquatic product, a decision still requires a defined tissue, analyte, method, consumption scenario, and uncertainty. Harmful blooms can also have nutritional and livelihood consequences, as illustrated for the Quinault fishery [144] and discussed in reviews of human dimensions and economic effects [145,146]. These consequences do not negate a toxicological concern, but they make the scope and duration of an advisory important.

California and French guidance provide examples of values derived for edible tissues [147,148]. The Victorian documents require attention to the analyte definition and document version [149,150]. In contrast, EU and U.S. EPA values concern drinking water [151,152], as does the Chinese GB 5749 standard [153]. The Chinese GB 5009.273 standard specifies determination of MCs in aquatic products rather than a maximum concentration [154]. Codex provides a general contaminant framework but does not establish an MC maximum level for aquatic products in the version examined here [155].

**Table 4:** Regulatory, advisory, and analytical frameworks relevant to microcystins in aquatic products.

Source and Date	Analyte and Matrix	Value and Reporting Basis	Exposure Assumptions	Status and Interpretation
California OEHHA, 2012 [147]	Microcystins LA, LR, RR, YR Edible sport fish and shellfish tissue	10 ng/g wet weight	32 g/day (=one 8 oz uncooked meal/week); 70 kg adult; 100% absorption assumed	Suggested action level derived for health protection; nonbinding. Product-specific tissue value; derived by OEHHA risk assessment.
France Afssa, 2008 [148]	Microcystins Edible fish tissue	5.6 µg/kg adults; 1.4 µg/kg children	Adults: 60 kg, 86 g fish/day + 2 L water/day at 1 µg/L; children: 10 kg, 57 g/day + water contribution	Health agency guidance derived from risk assessment; not a statutory food ML. Primary French health agency opinion; values depend on the exposure scenario.
Victoria Department of Health webpage (updated 27 December 2025) and linked 2011–12 factsheet [149]	Microcystin-LR or equivalent hepatotoxins; the current webpage presents these values under nodularin guidance Whole organism sample	Fish 24; prawns 32; mussels or mollusks 51 µg/kg whole organism	Seafood health risk assessment specific to the jurisdiction	Guidance and advice for the specified area. The linked factsheet defines the analyte as “Microcystin-LR or equivalent toxins, i.e., Nodularin”; interpretation depends on the cited document version, method, and reporting basis.
WHO Guidelines for drinking water quality, 2026 [143]	Total microcystins Drinking water	1.0 µg/L lifetime; 12 µg/L short term	Ingestion of drinking water	Nonbinding guideline for health protection. Not a residue limit for aquatic products.
Directive (EU) 2020/2184 [151]	MC-LR Water intended for human consumption	1.0 µg/L	Drinking water; monitored when blooms may be present	Binding parametric value after national transposition. Not a food marketability criterion.
U.S. EPA health advisories, 2015 [152]	Microcystins Drinking water	0.3 µg/L young children; 1.6 µg/L older children/adults for 10 days	Short-term exposure through drinking water	Nonregulatory advisory. Not a seafood residue value.
GB 5749-2022 [153]	MC-LR Drinking water	1.0 µg/L	Ingestion of drinking water	Mandatory Chinese national standard. Not a residue limit for aquatic products.
GB 5009.273-2016 [154]	MC-LR, MC-RR, MC-YR Aquatic products	Analytical method; no concentration limit	Not applicable	National food safety analytical method standard. Defines LC-MS/MS determination, not a maximum level.
FSA Lough Neagh, 2026 [32]	Free congeners; total MCs and nodularins Fish flesh, liver, GI tract, whole roach	Concentrations measured at the assessed site; no statutory ML	Fish consumption in defined scenarios and incomplete evisceration assumptions	Official risk assessment for the specified area. Transferable reasoning structure; numerical outcomes are not general limits.
Codex CXS 193-1995, rev. 2025 [155]	General contaminant framework Food and feed	No MC maximum level for aquatic products	Not applicable	International standard framework. Defines ML principles but no MC value for fish, shellfish or crustaceans.

Note. Product-specific values are separated from criteria for drinking water, standards for analytical methods, and assessments for defined sites. For Victoria, the current Department of Health webpage (updated 27 December 2025; accessed 1 September 2026) presents 24, 32, and 51 µg/kg as nodularin guideline values for fish, prawns, and mussels or mollusks. Its linked 2011–12 factsheet defines the analyte as “Microcystin-LR or equivalent toxins, i.e., Nodularin” and reports values in µg/kg of whole organism sample. Comparison across jurisdictions therefore requires the document version, analyte definition, analytical method, and result basis. LA, microcystin-LA; LR, microcystin-LR; RR, microcystin-RR; YR, microcystin-YR; GI, gastrointestinal; MC, microcystin; ML, maximum level.

### 6.1. Maximum Levels and Action Thresholds for Aquatic Products

Existing product-specific guidance demonstrates that the absence of a harmonized statutory maximum level is not equivalent to an absence of values based on tissue concentrations. California OEHHA proposed a 10 ng/g action level on a wet weight basis for microcystins in edible sport fish and shellfish, while the French Afssa opinion derived values that depend on the exposure scenario for edible fish tissue [147,148]. A regulatory comparison must therefore specify the product category, consumed tissue, basis in wet or dry weight, analyte definition, accepted method, toxicological point of departure, consumption scenario, legal effect, and applicable year. Water guidance cannot be converted into a food concentration without an exposure calculation for the specific product [7].

Where a jurisdiction lacks a statutory ML for aquatic products, authorities may still use explicitly labeled action levels, guidance values, or decision rules for a defined site. Such tools require product sampling, matrix-validated screening and confirmation, consumption scenarios, and a defined legal consequence for exceeding a decision value. General fishery guidance on hazard control [156] is a useful procedural analogy for documentation, verification, and corrective action but does not establish an MC threshold. Screening values prioritize samples, whereas market actions require stated confirmation and exposure evidence.

Codex CXS 193-1995, revised in 2025 [155], provides general principles for contaminant MLs but no microcystin ML for aquatic products. Product-specific distinctions among edible muscle, products consumed whole, and foods that include viscera are therefore jurisdictional management choices rather than existing Codex requirements. The Victorian source pair illustrates the need for interpretation specific to the document version: the current webpage presents the 24, 32, and 51 µg/kg values under nodularin guidance, whereas the linked factsheet defines the analyte as “Microcystin-LR or equivalent toxins, i.e., Nodularin” [149,150].

A central technical barrier is the analyte definition. A congener sum depends on which standards are available, whereas oxidation estimates a broader burden without retaining the mixture profile [48]. Proposed product thresholds must therefore specify both the measurement and its toxicological interpretation. General guidance on dose–response modeling can inform hazard assessment [157] but does not supply missing oral potency data for MC congeners or bound fractions.

### 6.2. Official Risk Assessments for Specific Products as Interim Models

The FSA Lough Neagh assessment provides a direct example of an interim approach for MCs in fish: it distinguishes edible flesh from other tissues, examines preparation and consumption assumptions, and evaluates uncertainty [32]. Marine biotoxin management offers procedural experience in monitoring and harvest controls [158] but cannot supply a numerical MC threshold by analogy. The transferable feature is a documented path from samples and assumptions to an advisory, not the numerical outcome of another toxin assessment.

Official assessments work best when responsibilities and update criteria are explicit. Environmental surveillance can initiate product-specific testing; laboratories establish the residue result; risk assessors interpret the relevant exposure scenario; and competent authorities communicate and periodically review the action. This allocation supports reversible management by tying surveillance, residue confirmation, exposure interpretation, communication, and periodic review to defined responsibilities.

### 6.3. Communication and Decision Thresholds

We recommend that an advisory identify the affected product or tissue, geographic area, consumers, duration, and conditions for review. Persistence during mussel depuration [30] and changes during fish preparation [47] support explaining why a clear water surface or routine cooking is not proof of safety. Drinking water management guidance illustrates how actions can be linked to monitoring and review [159]. Deep cyanobacterial layers [160] and limitations of satellite estimates [161] further justify stating what an environmental trigger can and cannot detect.

### 6.4. Proposed Framework Linking Evidence to Governance

The proposed framework addresses a recurring discontinuity: environmental monitoring identifies potential contamination events, whereas product decisions require evidence matched to the consumed matrix and decision consequence. Figure 3 links these domains while preserving the distinction between screening, confirmatory quantification, total burden analysis, and exposure interpretation. Its purpose is to identify when additional evidence is required, not to prescribe a universal threshold.

Implementation requires predefined environmental triggers, representative product sampling, matrix-validated methods, confirmatory capacity, an explicit consumption scenario, legal authority, and review and release criteria. The decision record needs to identify the product and tissue, analyte or fraction, concentration basis, exposure scenario, rationale, action, responsible authority, and principal uncertainty. The framework is a proposal to support decisions rather than a standardized regulatory protocol.

## 7. Current Challenges and Research Directions

The following research priorities are recommendations derived from the evidence gaps identified in this review. To avoid implying policy precision that the literature does not support, they are organized as priorities for immediate, intermediate, and long time horizons rather than fixed implementation deadlines.

Priority 1: comparable occurrence data. We recommend reporting species, life stage, harvest location, bloom conditions, edible portion, analytical fraction, congener panel, original mass basis, reporting limits, and recovery. Reviews of analytical capacity [162] and bloom monitoring [163] support the need for accessible, interpretable data. Tissue concentrations can then be assembled into regional datasets without pooling incompatible records.

Priority 2: laboratory comparability. Validation in fish [66], shellfish [68], and mussel reference material development [88] provides a starting point for interlaboratory trials. We recommend blinded testing of muscle, organ, and whole soft tissue matrices, with predefined criteria for recovery, precision, identification, and quantification. The goal is comparable performance across laboratories, not merely lower detection limits in solvent.

Priority 3: biological relevance of the additional burden. The difference between free and total measurements in tilapia [25] and the methodological issues reviewed by Bouteiller et al. [48] justify paired chemical and biological studies. Analysis of the same tissue by targeted LC-MS/MS, oxidation, digestion or bioaccessibility methods, and toxicity assays could establish whether the additional fraction changes internal dose. Bound fractions require this evidence before incorporation into an oral potency scheme.

Priority 4: thresholds for defined products. Existing tissue guidance [147,148] and congener toxicology [106] show why a candidate threshold needs both an exposure scenario and an analyte definition. We recommend developing testable options for edible muscle, whole organisms, and foods containing viscera separately. A universal value would conceal differences that remain unresolved in the evidence.

Priority 5: prospective evaluation of management. Nutrient enrichment research [164] and satellite forecasting studies [165] support prevention and surveillance, but the effect of these tools on product decisions needs direct evaluation. We recommend testing sampling triggers, analytical turnaround, temporary restrictions, and release criteria in fisheries with contrasting exposure and consumption patterns. This would establish whether the proposed framework improves protection while keeping restrictions proportionate.

## 8. Conclusions

This review links chemical and tissue evidence to consumer risk by retaining the distinctions among the sampled organ, measured fraction, mass basis, and exposure scenario. Quantitative examples show why a congener measurement, a total burden estimate, and a screening response cannot be used interchangeably. Algal supplements add a separate exposure pattern because concentrated products may be taken repeatedly in small doses. The evidence remains insufficient for a universal food limit or a complete oral potency scheme for MC mixtures. Priority actions are comparable residue data, validated methods in edible matrices, characterization of bound fractions after ingestion, and prospective testing of explicit decision criteria.

## Figures and Tables

**Table 1:** Selected foundational and recent reviews relevant to microcystins in aquatic foods.

Review	Design or Emphasis	Principal Scope	Product Relevance	Relation to This Review
Ibelings and Chorus [15]; Pham and Utsumi [16]	Narrative synthesis	Accumulation in freshwater organisms and ecosystems	Fish and other freshwater foods	Basis for separating environmental transfer from consumer intake
Drobac Backović and Tokodi [4]; Drobac Backović and Tokodi [7]	Narrative synthesis	Cyanotoxins in food and freshwater aquaculture	Food exposure and aquaculture hazards	Context for tissue, product, and consumption distinctions
Melaram et al. [11]; Rajpoot et al. [12]	Health and mechanism reviews	MCs, nodularin, and MC-LR toxicology	Hazard and exposure routes	Mechanisms require separate residue and intake evidence
Fraga et al. [8]	Systematic review	Freshwater origins and marine consequences	Transfer into transitional and marine settings	Environmental transfer is linked here to product decisions
Withana et al. [13]	Narrative synthesis	Microalgal toxins in food	Algal foods and nutritional products	Supports a distinct assessment of dietary supplements
Shao et al. [10]	Narrative synthesis	Food chain transfer, detection, and detoxification	Food contamination pathways	Shared scope; this review emphasizes compatible decision inputs
Moon et al. [14]	Systematic review and meta-analysis	Chronic hepatic, renal, and metabolic outcomes	Human hazard evidence; not seafood attribution	Human associations do not replace dietary exposure data
Present review	Critical narrative and framework review	Compatibility of occurrence, methods, hazard, and exposure	Edible matrices and algal supplements	Explicit assumptions connecting residue evidence to management

Note. The comparison describes the principal scope of each review, not a quality ranking or a claim that any topic was entirely absent. PRISMA, Preferred Reporting Items for Systematic Reviews and Meta-Analyses.

**Table 3:** Analytical methods and decision roles for microcystin monitoring in aquatic products.

Method	Primary Role	Minimum Matrix Validation	Suggested Decision Role Under Stated Validation Conditions	Key Limitation
LC-MS/MS [66,67,68]	Targeted quantitative confirmation	Calibration matched to the matrix, recovery, retention time, diagnostic transitions, internal standard strategy, and batch QC	Primary quantitative confirmation when matrix and analyte panel are validated	Limited to calibrated analytes and standards.
LC-HRMS [69,70]	Suspect and untargeted screening; confirmation only when identification and calibration criteria are met	Accurate mass, isotope pattern, fragmentation, retention time evidence when standards exist, matrix controls, and transparent identification confidence levels	Supports confirmation only with reference standards, calibration, and predefined identification confidence	Tentative features are not equivalent to validated quantitative results.
ELISA or immunoassay [71,72]	Screening and sample prioritization	Dilution or cleanup, spike recovery, cutoff specific to the matrix, assessment of false results, and plan to confirm positive results	Surveillance screening	Cross reactions and matrix effects; insufficient alone for market action.
PP1/PP2A inhibition assay [73,74]	Functional inhibition screening	Controls for matrix inhibition unrelated to MCs, reference activity, and recovery	Supporting or research evidence	Does not identify congeners or analytical fractions.
MMPB after Lemieux oxidation [75,76,77]	Estimate combined free and bound burden containing Adda	Oxidation recovery, blanks, calibration, matrix QC, and interpretation controls	Decision support when additional burden containing Adda is relevant	Loses congener identity and is not interchangeable with targeted results for free congeners.
Portable biosensors and aptasensors [78,79]	Early warning and field triage	Independent validation in the target product or process water matrix and a laboratory confirmation pathway	Prioritization before a decision	Generally requires laboratory confirmation before product action.

Note. Suggested decision roles are assigned by the authors on the basis of validation in the target matrix, analyte identification confidence, quantitative calibration, batch quality control (QC), and the need for independent confirmation; they are not regulatory method classifications. Abbreviations: Adda, (2S,3S,8S,9S)-3-amino-9-methoxy-2,6,8-trimethyl-10-phenyldeca-4,6-dienoic acid; ELISA, enzyme-linked immunosorbent assay; LC-HRMS, liquid chromatography coupled with high resolution mass spectrometry; LC-MS/MS, liquid chromatography tandem mass spectrometry; MMPB, 2-methyl-3-methoxy-4-phenylbutyric acid; PP1/PP2A, protein phosphatases 1 and 2A; QC, quality control.

## Data Availability

No new experimental data were generated. Supplementary Table S1 documents the search scope, historical limitations, and reproducible strategies. Supplementary Table S2 reports the records and publication year counts from the PubMed searches performed on 2 October 2026. The search archive contains the corresponding queries, PMID lists, and retrieved records. Historical database exports and selection counts were not retained and have not been reconstructed. Evidence supporting individual scientific statements is available in the cited literature and official sources.

## Supplementary Materials

The following supporting information can be downloaded at: https://www.mdpi.com/article/10.3390/toxins18100439/s1. Table S1, Search scope and reproducible update strategies; Table S2, Documented PubMed update and publication year profile; Supplementary search archive, queries, PMID lists, and retrieved records from 2 October 2026.

## Abbreviations

Adda	Characteristic β-amino acid of microcystins; full chemical name in Section 1.3
AOP	Advanced oxidation process
bw/BW	Body weight/body weight variable in exposure equations
dw	Dry weight or dry mass
EDI	Estimated daily intake
ELISA	Enzyme-linked immunosorbent assay
EU	European Union
FAO	Food and Agriculture Organization of the United Nations
FSA	Food Standards Agency
GI	Gastrointestinal
IAEA	International Atomic Energy Agency
IOC	Intergovernmental Oceanographic Commission
IR	Ingestion rate
LC-HRMS	Liquid chromatography coupled with high resolution mass spectrometry
LC-MS/MS	Liquid chromatography coupled with tandem mass spectrometry
LOQ	Limit of quantification
MC/MCs	Microcystin/microcystins
MC-LR	Microcystin with leucine and arginine at positions 2 and 4
Mdha	N-methyldehydroalanine
ML	Maximum level
MMPB	2-Methyl-3-methoxy-4-phenylbutyric acid
OATP	Organic anion transporting polypeptide
OEHHA	Office of Environmental Health Hazard Assessment
PDB	Protein Data Bank
PMID	PubMed identifier
PP1/PP2A	Protein phosphatase 1/protein phosphatase 2A
PRISMA	Preferred Reporting Items for Systematic Reviews and Meta-Analyses
QC	Quality control
TDI	Tolerable daily intake
TEF/TEQ	Toxic equivalency factor/toxic equivalent
U.S. EPA	United States Environmental Protection Agency
WHO	World Health Organization
ww	Wet weight
