# Supplementary material for: Microcystins in Aquatic Food Products: Linking Environmental Transfer, Consumed Matrices, and Dietary Risk
Source: Toxins (Basel). 2026 Oct 12;18(10):439. doi: 10.3390/toxins18100439 (PMC13709362; doi:10.3390/toxins18100439)
Supplement: Supplementary file 1 [file toxins-18-00439-s001.zip › toxins-4587466-supplementary.pdf]

Supplementary Materials: Microcystins in Aquatic Food Products: Linking Environmental Transfer, Consumed Matrices, and Dietary Risk

Xiaoyu Xiao, Junxuan Zeng, Songheng Zheng, Shifeng Liu, Qi Deng, Mei Qiu, Zhijia Fang, Gaoshuang Hu, Yaling Wang, Youxin Wang

Table S1. Search scope and reproducible update strategies.

The original review was a critical narrative and framework review. The entries below distinguish the reported search history, strategies retained for reproducible updates, and searches actually executed for this revision. Only the revision update has retained electronic retrieval records; no historical selection counts are reconstructed.

Stage or field	Documented scope and limitation
Original search	Web of Science Core Collection, Scopus, PubMed, Crossref, and OpenAlex; inception through 18 June 2026. Original exports and database provenance of selected articles were not retained.
Presubmission update	8 August 2026: PubMed, Crossref, OpenAlex, publisher platforms, and official sources. Web of Science and Scopus were not exported again. Historical counts remain unavailable.
Revision update	2 October 2026: three PubMed searches using ESearch and EFetch. All returned PMIDs and bibliographic records were retained. Separate publisher and official source checks verified specific claims but are not treated as counted database searches.
Language	No language filter in the revision queries. Use in the narrative required sufficient accessible information for appraisal; availability can introduce language and access bias.
Inclusion	Aquatic organisms, foods, or algal supplements with interpretable tissue or product information, analytical fraction, concentration basis, method, exposure estimate, or management relevance. Recent reviews relevant to chemistry, toxicology, food exposure, or analysis were considered.
Exclusion from product conclusions	Terrestrial food studies; duplicate datasets; conference abstracts without adequate data; outcomes unrelated to MCs; insufficient methodological detail. Water studies support only environmental or treatment interpretation.
Extracted fields	Author and year; DOI or PMID; species and product; tissue; location and design; analyte panel or fraction; method; concentration and original unit/basis; reporting limit; exposure assumptions and uncertainty.
Deduplication	Revision update: unique PMID; references additionally checked by DOI. The three query yields overlap and must not be added to obtain a unique total.
Interpretation	The revision search supports a focused narrative update, not a complete systematic review. Cited records in Table S2 include previously cited studies as well as additions; these counts are not historical selections.

Strategies retained for future updates

The following topic blocks and field syntax preserve the earlier S1 strategy. They are reproducible proposals for an update, not verified transcripts of the unretained June search. The dated searches actually run for this revision are given separately below.

A: microcystin* OR "microcystin-LR" OR MC-LR OR nodularin*

B: fish OR finfish OR shellfish OR bivalv* OR mussel* OR oyster* OR clam* OR crustacean* OR shrimp OR prawn* OR crayfish OR "aquatic food*" OR seafood

C: bioaccumulat* OR tissue OR edible OR residue* OR exposure OR intake OR risk OR detect* OR analys* OR screen* OR confirm* OR processing OR cooking OR depuration OR guideline* OR regulation*

Web of Science Core Collection: TS=(A) AND TS=(B) AND TS=(C). Scopus: TITLE-ABS-KEY(A) AND TITLE-ABS-KEY(B) AND TITLE-ABS-KEY(C). Expand A, B, and C verbatim before execution; record the execution date, publication date range, language limits, query, and export counts. These databases were not searched again for this revision.

PubMed: expand the same blocks with [Title/Abstract] on each term and join the parenthesized blocks with AND; append a stated publication date interval. The original broad PubMed strategy is retained as a future update option, whereas the three focused queries below are the executed revision searches.

Crossref and OpenAlex were used historically for bibliographic or metadata checks with combinations of the topic terms. No exact historical query transcript, ranked results, or selected-record provenance is available. They are not represented here as reproducible counted searches. Publisher DOI pages and official agency documents were used to verify authorship, versions, analyte definitions, and numerical statements.

PubMed queries executed on 2 October 2026

Recent reviews (68 records)
microcystin*[Title/Abstract] AND (review[Publication Type] OR review[Title]) AND ("2024/01/01"[Date - Publication] : "2026/10/02"[Date - Publication])

Algal supplements (198 records)
microcystin*[Title/Abstract] AND ("dietary supplement*"[Title/Abstract] OR "food supplement*"[Title/Abstract] OR "algal supplement*"[Title/Abstract] OR Aphanizomenon[Title/Abstract] OR spirulina[Title/Abstract]) AND ("1900/01/01"[Date - Publication] : "2026/10/02"[Date - Publication])

Recent tissue studies (21 records)
microcystin*[Title/Abstract] AND (fish[Title/Abstract] OR shellfish[Title/Abstract] OR bivalv*[Title/Abstract] OR shrimp[Title/Abstract] OR crayfish[Title/Abstract]) AND (tissue[Title/Abstract] OR edible[Title/Abstract] OR muscle[Title/Abstract]) AND ("2024/01/01"[Date
- Publication] : "2026/10/02"[Date - Publication])

The searches applied no language filter. The two recent-literature queries cover 1 January 2024 to 2 October 2026; the supplement query covers 1 January 1900 to 2 October 2026. The broad supplement terms also retrieve ecological and taxonomic studies. Retrieval is therefore not equivalent to eligibility. Exact query translations and PMID lists are retained in the accompanying archive.

Table S2. Documented PubMed update and publication year profile.

The three queries returned 287 records before removal of overlap and 282 unique PMIDs. Of these, 21 records are cited in the revised manuscript, identified by exact DOI matching. This column includes studies already present in the original reference list. It is not the number of newly added articles and is not a reconstruction of the original database screening. Publication years use the journal publication year in the retrieved PubMed record, which may differ from an online publication year.

Year	Reviews query	Supplements query	Tissues query	Unique PMIDs	Cited in revision
1992	0	1	0	1	0
1996	0	1	0	1	0
1997	0	1	0	1	0
1998	0	1	0	1	0
1999	0	1	0	1	0
2000	0	1	0	1	0
2001	0	3	0	3	0
2002	0	3	0	3	0
2003	0	3	0	3	0
2004	0	1	0	1	0
2005	0	7	0	7	1
2006	0	7	0	7	0
2007	0	5	0	5	0
2008	0	6	0	6	0
2009	0	4	0	4	0
2010	0	4	0	4	0
2011	0	8	0	8	0
2012	0	8	0	8	0
2013	0	6	0	6	0
2014	0	7	0	7	0
2015	0	11	0	11	0
2016	0	3	0	3	0
2017	0	6	0	6	0
2018	0	13	0	13	1
2019	0	12	0	12	0
2020	0	7	0	7	1
2021	0	13	0	13	0
2022	0	11	0	11	1
2023	0	10	0	10	0
2024	27	14	5	43	6
2025	23	12	12	46	7
2026	18	7	4	29	4
Total	68	198	21	282	21

The same article may occur in more than one query column. The unique and cited columns are deduplicated. Zero denotes no retrieved record for that year/query; it does not establish absence of research.

Historical database attribution

Source	Original selected articles by publication year
Web of Science Core Collection	Not recoverable: original export and source attribution were not retained.
Scopus	Not recoverable: original export and source attribution were not retained.
PubMed before the revision update	Not recoverable; the documented October update is reported separately.
Crossref and OpenAlex	No retained historical query transcript or selected-record attribution.
Publisher and official sources	Targeted verification, not a counted database selection process.
